# Persistent calyces increase floral longevity and female fitness in *Salvia miltiorrhiza* (Lamiaceae)

**DOI:** 10.1093/aobpla/plac004

**Published:** 2022-01-27

**Authors:** Deng-Fei Li, Yan Yu, Hao-Jin Yang, Xian-Chun Yan

**Affiliations:** 1 Key Laboratory of Southwest China Wildlife Resources Conservation (Ministry of Education), China West Normal University, Nanchong 637002, China; 2 School of Life Sciences, Central China Normal University, Wuhan 430079, China

**Keywords:** Fruit set, nectar robber, persistent calyx, pollination, *Salvia*, seed mass

## Abstract

The evolution of persistent calyces may be an adaptation to ensure reproductive success of certain flowering plants. However, experimental evidence of the functions of persistent calyces during flowering and seed development remains scarce. We explored the possible functions of persistent calyces in *Salvia miltiorrhiza*, a perennial herb with campanulate calyx. We conducted calyx manipulation experiments to examine whether persistent calyces affect visitation rates of nectar robbers and pollinators, individual flower longevity, fruit set, seed set and seed mass. Our findings suggested that shortening of the calyx significantly decreased individual flower longevity, fruit set and seed mass, but did not affect visitation of pollinators and nectar robbers. In addition, the seed set of control flowers and the flowers with calyx shortened at the beginning of fruiting stage (CSF flowers) did not significantly differ, but both were higher than that of the flowers with calyx shortened at the beginning of blooming stage (CSB flowers). The seed set and fruit set of CSB flowers were limited by pollination due to the reduction in floral longevity. We conclude that persistent calyces of *S. miltiorrhiza* may represent adaptive strategies to maintain floral longevity and increase plant fitness. Persistent calyces may provide protection for the growth of flowers and contribute resources to the development of fruits and seeds.

## Teaser

Floral traits mainly function during flowering, while the calyces of some plant species persist well beyond completion of anthesis, often until fruit maturation. We conducted calyx manipulation (shortening of the calyx) experiments to explore the possible functions of persistent calyces in *Salvia miltiorrhiza*, a perennial herb with a campanulate calyx. Our results suggested that persistent calyces of *S. miltiorrhiza* may provide protection for the growth of flowers after they have emerged fully from the buds and contribute resources (such as carbon, energy, etc.) to the development of fruits and seeds.

## Introduction

Many flowering plants have evolved distinctive floral traits to ensure reproductive success (Lloyd and Barrett 1996; Song *et al.* 2013; Sun and Huang 2015). The evolution of floral traits is shaped by various selection forces, including pollinators, biotic non-pollinators and abiotic factors (Waser and Price 1983; Nilsson 1988; Herrera 1997; Herrera *et al.* 2003; Strauss and Whittall 2006; Sun *et al.* 2008; Yang and Sun 2009; Li *et al.* 2021). For example, Wang *et al.* (2013) showed that pollinators favoured flowers with large spur circle in *Impatiens oxyanthera*. Mccarren *et al.* (2021) found that corolla stickiness protects *Erica* flowers against nectar robbers. Floral traits mainly function during flowering, while the calyces of some plant species persist well beyond completion of anthesis, often until fruit maturation (Herrera 2005; Wang *et al.* 2010). However, the evolutionary significance of persistent calyces during flowering and/or seed development in flowering plants is still poorly understood.

Previous studies have partially revealed the adaptive value of calyces related to pollination during flowering (Fukuda *et al.* 2001; Dohzono and Suzuki 2002; Dohzono *et al.* 2004). For example, Dohzono *et al.* (2004) examined the adaptive significance of a temporal decrease in the calyx tube length of *Clematis stans*, a dioecious species pollinated by *Bombus diversus* (with long proboscis) and *B. honshuensis* (with short proboscis). They showed that *C. stans* produce different types of flowers with long (male) and short (female) calyx tubes, which may be an adaptive strategy when the pollinator fauna fluctuates. In addition, Fukuda *et al.* (2001) showed that the arch structure formed by lateral sepals functions to ensure correct pollination in *Aconitum japonicum*. There are many plant genera in which the calyx sepals take on all or part of the roles of advertisement and pollinator attraction (Willmer 2011). Attractive calyces may act in concert with petals, whether of the same colour (e.g. *Salvia*) or contrasting colours (Willmer 2011). The calyces as advertising structures increase the showiness of the whole floral display.

Calyx sepals can be formed from a thickened epidermis that is relatively solid and free of airspaces or can be toughened by sclerenchyma to give a semiwoody protective base to the flower (Willmer 2011). We hypothesized that calyces may help to provide protection for flower growth and maintain floral longevity. Furthermore, protection against nectar robbers may have also played an important role in the evolution of calyces (Roubik 1982; Inouye 1983; Irwin 2010). Plants may avoid nectar robbing by producing flowers with thick and long calyces, making it mechanically difficult for nectar robbers to make holes to access the nectar (Inouye 1983; Irwin 2010). However, these hypotheses (i.e. advertisement and protection roles of calyces) have not been tested.

Moreover, the protracted presence of calyces during flowering suggests that they have additional functions unrelated to pollination after anthesis (Sisterson and Gould 1999; Herrera 2005; Wang *et al.* 2010). The chlorophyll-containing sepals may contribute carbon and energy requirements to the development of fruits through photosynthetic capacity (Williams *et al.* 1985; Vemmos and Goldwin 1994; Smillie *et al.* 1999; Herrera 2005). In some plant species, photosynthetic activity of green sepals at the flowering or early post-flowering stages may be comparable or even exceed that of leaves (Williams *et al.* 1985; Vemmos and Goldwin 1994; Smillie *et al.* 1999; Herrera 2005). Based on previous studies, we hypothesized that persistent calyx may increase plant fitness during seed development (Vemmos and Goldwin 1994; Herrera 2005; Wang *et al.* 2010). For example, Vemmos and Goldwin (1994) reported that removal of the sepals significantly reduced fruit setting in *Molus pumila*. Herrera (2005) provided experimental evidence that the green, persistent calyx of *Helleborus foetidus* contributed resources to the development of seeds, and that removing the calyx reduced seed mass by an average of about 10 %. However, calyx manipulation had no significant effect on seed set or number of seeds in this species. In addition, Wang *et al.* (2010) found that water-filled calyx could facilitate the development of fertilized ovules during fruiting in *Anisodus luridus*. However, seed number, seed set and seed mass of fruits that had water removed from the calyx were reduced greatly. Calyces could increase plant fitness via different mechanisms across plant species. Therefore, further experimental evidence in different plant species are required to test the hypothesis that persistent calyx may increase plant fitness during seed development.

Here, we conducted calyx manipulation experiments to test the hypotheses related to how persistent calyces benefit plants during flowering and seed development in *Salvia miltiorrhiza*. This species is distributed in many areas of north and south China. Pollinators and nectar robbers visit the flowers, and the campanulate calyces persist until the seeds have ripened. Based on field investigations, we aimed to address the following questions: do persistent calyces (i) enhance pollinator visitation, (ii) provide protection against nectar robbers, (iii) increase floral longevity and (iv) increase the number of fruits and seeds.

## Materials and Methods

### Study site and species

**Figure 1.**
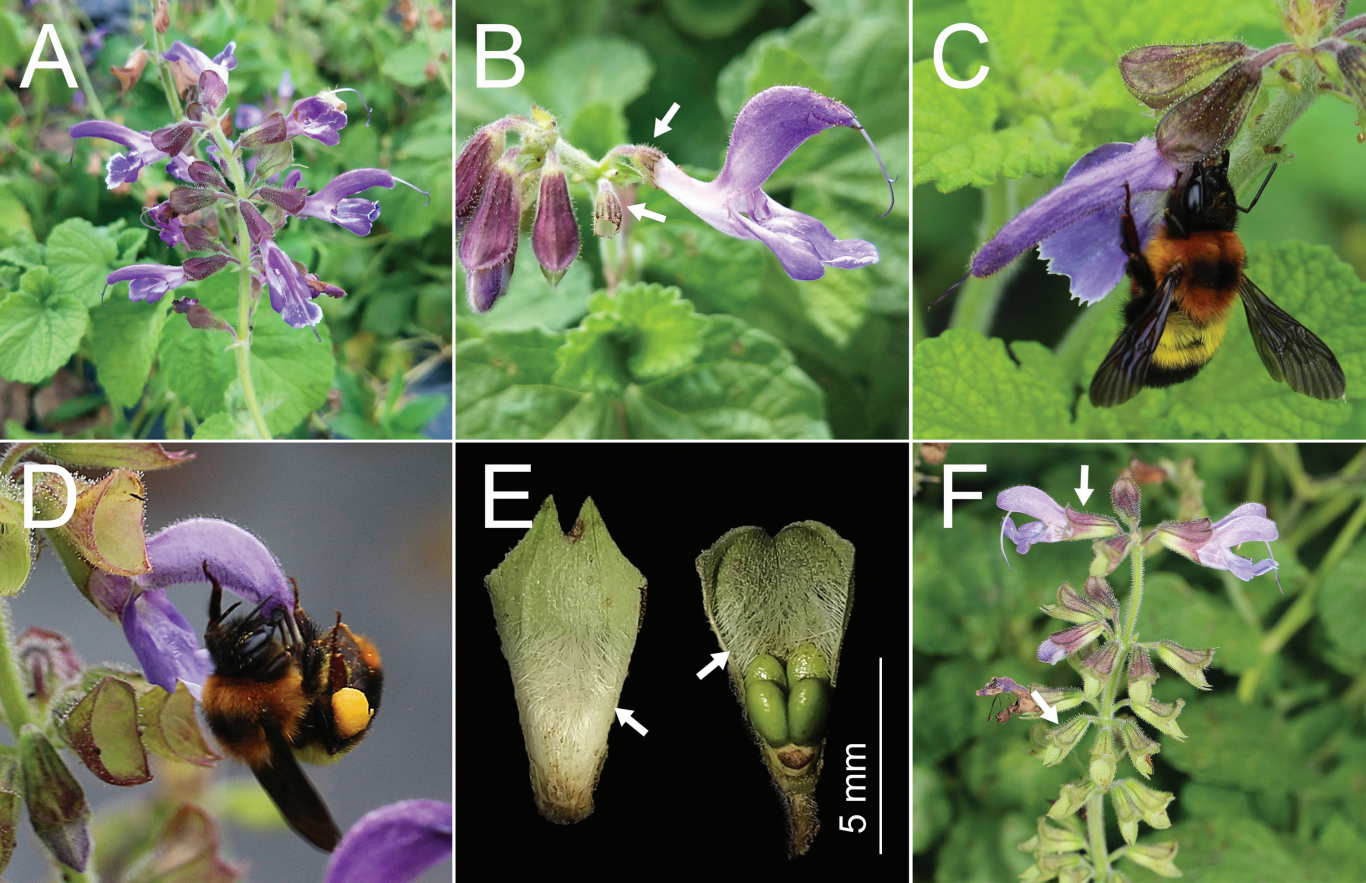
Calyces in *Salvia miltiorrhiza* and floral visitor species. (A) An inflorescence with verticillasters; (B) a shortened calyx of flower, the shortened calyx marked with white arrows; (C) *Bombus breviceps* acting as a nectar robber; (D) *B. breviceps* as a pollinator; (E) a vertical section of campanulate calyx, the calyx tube with a hairy annulus on throat inside, the densely white hairs marked with white arrows; (F) an inflorescence with purple (floral stage) and green (post-floral stage) campanulate calyces marked with white arrows.

This study was conducted during the flowering season of 2021 in the experimental field of China West Normal University (30°48′N, 106°04′E; 270 m above sea level) in northeast Sichuan, Southwest China. *Salvia miltiorrhiza* (Lamiaceae) is a perennial herb distributed in many areas of both north and south China. It inhabits hillsides, meadows and forests. The study area was approximately 10 m × 20 m and included at least 200 *S. miltiorrhiza* individuals, located on a slope. Flowering generally occurs from May to early August. Flowering individuals can grow up to 0.8 m high and produce numerous vertical inflorescences with verticillasters (Fig. 1A). Flowers are highly zygomorphic, blue-purple or purple, with a hooded upper lip. The style is exserted and protrudes out of the upper lip. Two upper stamens are fertile and hidden below the upper lip, while two lower stamens are mostly reduced and act as a barrier on the pollinator’s way to the nectar. Nectar is secreted at the ovary base and accumulates in the narrow corolla tube. More than half of the corolla tube is covered by a campanulate calyx, which persists well until fruit maturation. Each flower produces four ovaries.

### Nectar robbing and pollination

Our field surveys showed that *Bombus breviceps* was the only visitor species to *S. miltiorrhiza* during our experiments. Moreover, *B. breviceps* foragers collected both pollen and nectar, acting as both pollinators and nectar robbers in this plant species. To evaluate the proportion of nectar robbing of each inflorescence under natural conditions, 20 inflorescences (each with approximately five fresh flowers) were randomly selected from 17 individuals during the flowering season, and the number of robbed flowers of each inflorescence was recorded at 17:00 p.m. The robbed flowers were identified according to holes in the corolla tubes that were bitten by *B. breviceps* foragers. We calculated the proportion of nectar robbing by dividing the total number of flowers by the number of robbed flowers for each inflorescence. To determine whether *B. breviceps* foragers provide pollination services for the flowers of *S. miltiorrhiza*, 10 inflorescences (each with approximately five fresh flowers) were randomly selected from 10 individuals, and bagged with fine-mesh polyester bags before blooming in July 2021. When the flowers were in bloom, they were exposed to floral visitors. Then, the flowers were taken back to the laboratory after visited one time by *B. breviceps* foragers, which collected pollen from the flowers. Each stigma was cut off with thin-tipped forceps and was placed on a slide, and then pollen grains were counted under a light microscope as the amount of pollen deposition. Spontaneous autogamy (i.e. self-pollen deposition) does not occur in *S. miltiorrhiza*, because the style is exserted and protrudes out of the upper lip and two fertile stamens are hidden below the upper lip. Similarly, a previous study done by Chen *et al.* (2018) on *Salvia yunnanensis* provides evidence that spontaneous autogamy is non-existent in this species although it is self-compatible, and that its sexual reproduction depends on pollinators.

### Effects of shortened calyx on visitation by nectar robbers and pollinators

To detect the effects of shortened calyx on the visitation rates of nectar robbers and pollinators, at least 50 inflorescences of *S. miltiorrhiza* were selected randomly from more than 20 individuals and were bagged with fine-mesh polyester bags before blooming in July 2021. When the flowers bloomed, we selected 60 flowers from more than 15 inflorescences (each with 3–5 fresh flowers) to conduct manipulation experiments, before 08:00 a.m. on each day between 24 and 26 July 2021. A total of 180 flowers (60 flowers × 3 days) were used in two treatments, with a total of 24 h (8 h × 3 days) of observations conducted for each treatment. A small pair of scissors shortened the calyces of 30 flowers, while the remaining 30 flowers without any treatment were used as a natural control. The length of the intact calyces was 7.383 ± 0.161 mm (mean ± SE, *N* = 30). We shortened the length of the calyx of each flower to approximately 2.7 mm only (Fig. 1B) as the ovary at the base of the calyces may be hurt when the length of calyces is shortened excessively. All flowers were exposed to floral visitors after the treatments. Subsequently, the visitation rates of nectar robbers and pollinators (visits per flower per 30 min) were recorded during the periods of 08:00–12:00 a.m. and 14:00–18:00 p.m., respectively.

### Effects of shortened calyx on individual flower longevity

To examine the effects of shortened calyx on individual flower longevity, 60 floral buds were marked randomly from 20 individuals on 28 July 2021. When the flowers bloomed, the calyces of 30 flowers were shortened, while the remaining 30 flowers without any treatment were used as a natural control. The calyx treatment was the same as described above. The fates of these flowers were tracked by checking them at 3-h intervals during daylight hours (from 07:00 a.m. to 19:00 p.m.), during which we recorded the longevity (hours) of the flower, whether the corolla had withered or not. Individual flowers were checked until abscission.

### Effects of shortened calyx on fruit and seed production

To test the effects of shortened calyx on fruit and seed production, 180 floral buds were marked randomly from 20 individuals (each with more than two inflorescences). These individuals were different from the individuals described above. When the flowers were in bloom, we conducted three experimental treatments: (i) control treatment—60 flowers from 20 individuals without any treatment were marked as a natural control; (ii) CSB—the calyces of 60 flowers from 20 individuals were shortened at the beginning of blooming stage to test the effect of shortened calyx on both flowering and fruit/seed production, using the calyx treatment described above; (iii) CSF—the calyces of 60 flowers from 20 individuals were shortened at the beginning of fruiting stage to examine the effect of shortened calyx on fruit/seed production. Two weeks later, the ripened fruits produced by these flowers were harvested, and the seeds and undeveloped ovules in each fruit were counted. Fruit set was calculated as simple fruit number per aggregate fruit divided by total ovary number. Seed set was calculated as seed number per fruit divided by total number of seeds and undeveloped ovules, in which aborted fruits were not included. Meanwhile, the seeds were weighed to the nearest 0.1 mg using an electronic balance MS304TS/02 (Mettler-Toledo International, Inc., Shanghai, China).

### Statistical analyses

All statistical analyses were performed using the software R version 4.0.3 (www.r-project.org). The visitation rates of nectar robbers and pollinators were not normally distributed. Accordingly, we assessed the effects of treatments (i.e. control and calyx-shortened) on visitation rates of nectar robbers and pollinators using two-sample Wilcoxon tests (R function *wilcox.test()* in the *rstatix* package). To examine how the individual flower longevity (hours) differed between natural and calyx-shortened flowers, we fitted generalized linear mixed models (GLMMs) to individual flower longevity (Poisson distribution with a log link). We used the fixed effect of treatment (i.e. control and calyx-shortened) and the random effect of flower ID nested in plant ID. To test how the treatments influenced fruit and seed production, we fitted GLMM (binomial distribution with a logit link) separately to fruit set and seed set. For both analyses we used the fixed effect of treatment (i.e. control, CSB and CSF) and the random effect of flower ID nested in plant ID. We performed a generalized linear model (GLM) with Gaussian distribution and identity-link function to determine the effects of the treatments on seed mass (with seed mass as dependent variable, and treatment as a factor). Seed mass was log-transformed to have normal distributed data.

Generalized linear mixed models and GLM were conducted using R package of *lme4* (Bates *et al.* 2019) and *stats*, respectively. The statistical significance of effects was determined with type III Wald χ ^2^ ANOVA testes in the package *car* (Fox and Weisberg 2011). To compare the different treatment combinations in the analyses, contrasts of estimated marginal means (adjustment method: Tukey) were computed (*emmeans* R package; Lenth 2020).

## Results

### Effects of shortened calyx on visitation by nectar robbers and pollinators

**Figure 2.**
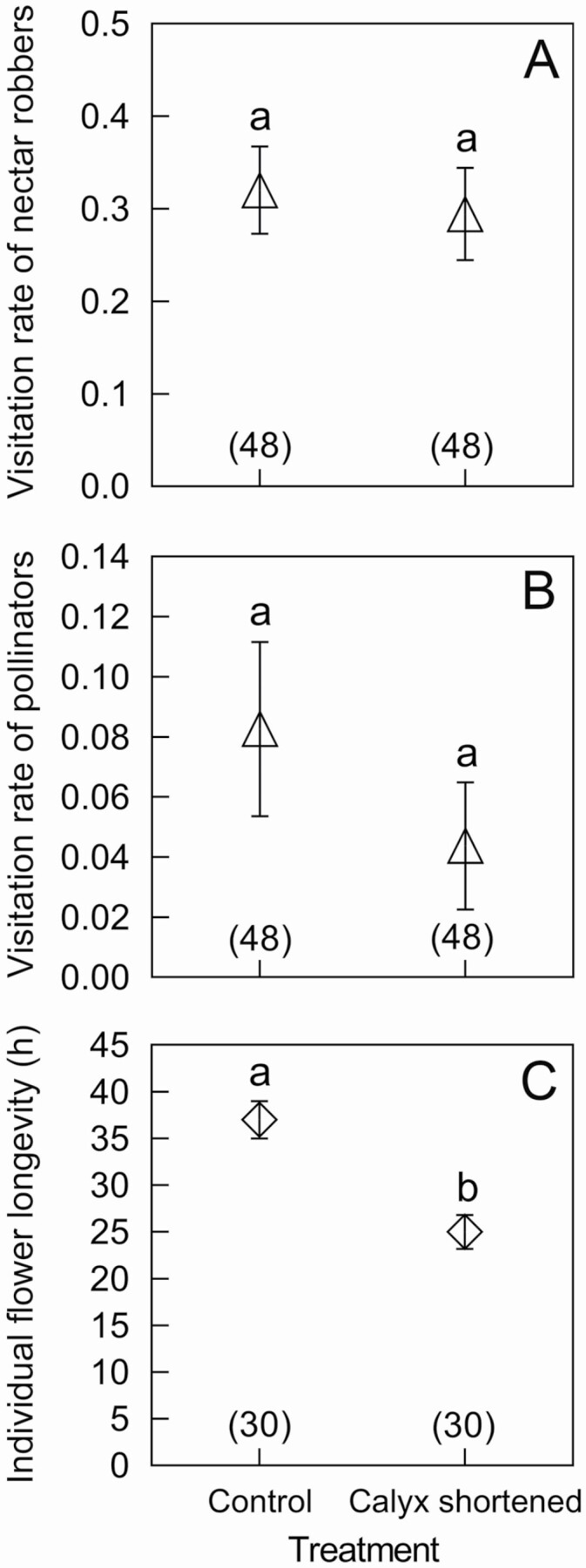
The effects of calyx treatments on visitation rates of nectar robbers (A) and pollinators (B) and individual flower longevity (C). Mean and standard errors (error bars) are presented. Different lowercase letters show significant differences at *P* < 0.05. The numbers in brackets represent sample size.

Pollinator and nectar robber visitation rates did not differ between flowers with control and shortened calyces (*Z* = −1.263, *P* = 0.207 and *Z* = −0.534, *P* = 0.593, respectively; Fig. 2A and B). We found that *B. breviceps* foragers acted as both nectar robbers and pollinators for *S. miltiorrhiza*. When a *B. breviceps* individual foraged for nectar, it acted as a nectar robber since it sucked nectar through a hole made on the corolla tube without touching the stamen or pistil of *S. miltiorrhiza* (Fig. 1C). The proportion of nectar robbing of each inflorescence under natural conditions was 88.881 ± 3.559 % (mean ± SE, *N* = 20). Moreover, we observed that *B. breviceps* did not enter the corolla tube to forage for nectar. When *B. breviceps* collected pollen from the upper lips of flowers, a large number of pollen grains were removed and deposited on its abdomen (Fig. 1D). Afterwards, it foraged for pollen on the next flower, and the abdomen contacted with the stigma and deposited pollen grains onto the receptive stigma. The pollen deposition per flower was 21 ± 5 (mean ± SE, *N* = 31) pollen grains in a single visit by *B. breviceps*. In this case, *B. breviceps* was identified as the effective pollinator of *S. miltiorrhiza*.

### Effects of shortened calyx on individual flower longevity

We found a significant effect of treatment on the individual flower longevity (GLMM, treatment χ ^2^ = 60.68, df = 1, *P* < 0.0001). The individual flower longevity of calyx-shortened flowers (25 ± 1.798 h, *N* = 30) was significantly lower than that of natural flowers (37 ± 2.015 h, *N* = 30, *P* < 0.0001; Fig. 2C). Calyx-shortened flowers withered and fell off earlier than natural flowers.

### Effects of shortened calyx on fruit and seed production

**Figure 3.**
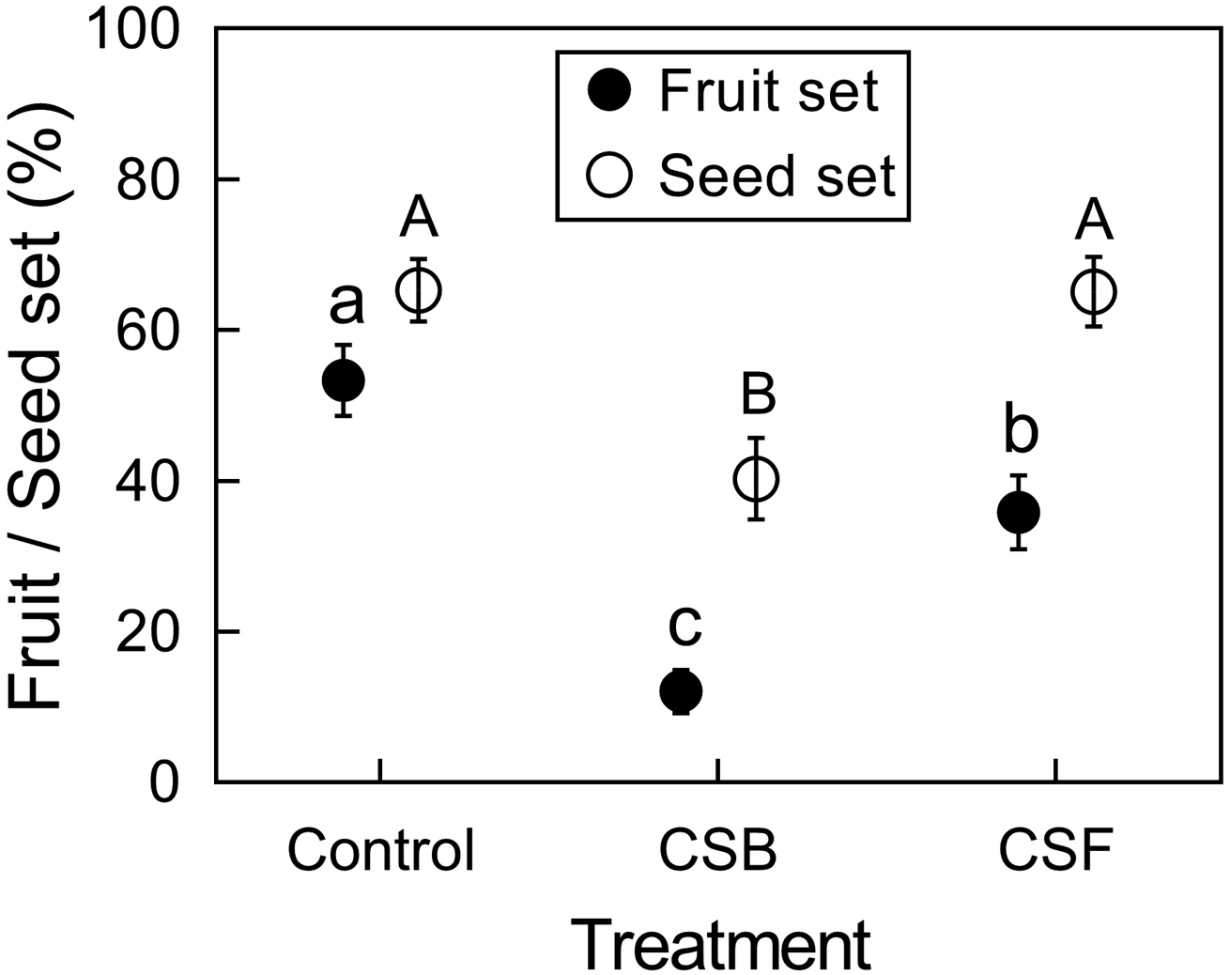
The effects of calyx treatments on fruit set and seed set. CSB, calyx shortened at the beginning of blooming stage. CSF, calyx shortened at the beginning of fruiting stage. Mean and standard errors (error bars) are presented. Different lowercase and capital letters, respectively, show significant differences at *P* < 0.05.

In control and shortened calyx treatments (i.e. CSB and CSF), 100 aggregate fruits and 243 seeds were harvested. Shortening of the calyx significantly affected both fruit set (GLMM, treatment χ ^2^ = 82.577, df = 2, *P* < 0.0001; Fig. 3) and seed set (GLMM, treatment χ ^2^ = 13.47, df = 2, *P* = 0.001; Fig. 3). The fruit set of control flowers was 53.33 ± 4.697 % (mean ± SE, *N* = 60), which was significantly higher than that of the CSB flowers (calyx shortened at the beginning of blooming stage, 12.08 ± 2.88 %, *N* = 60, *P* < 0.0001) and CSF flowers (calyx shortened at the beginning of fruiting stage, 35.83 ± 4.913 %, *N* = 60, *P* = 0.0006). The fruit set of CSF flowers was significantly higher than that of CSB flowers (*P* < 0.0001). Moreover, the seed set of control flowers (65.31 ± 4.112 %, *N* = 49) and that of CSF flowers (65.15 ± 4.608 %, *N* = 33) were not significantly different, but were both higher than that of CSB flowers (40.28 ± 5.4 %, *N* = 18, *P* = 0.002 and *P* = 0.003 for control and CSF flowers, respectively; **see****Supporting Information—Appendix S1**).

**Figure 4.**
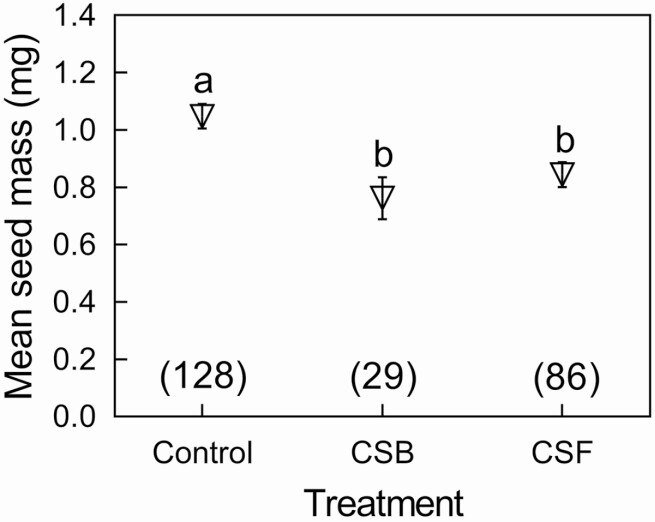
The effects of calyx treatments on seed mass. CSB, calyx shortened at the beginning of blooming stage. CSF, calyx shortened at the beginning of fruiting stage. Mean and standard errors (error bars) are presented. Different lowercase and capital letters, respectively, show significant differences at *P* < 0.05. The numbers in brackets represent sample size.

The results showed that shortened calyx significantly affected seed mass (χ ^2^ = 19.705, df = 2, *P* < 0.001; Fig. 4). The seed mass of control flowers was 1.048 ± 0.043 mg (mean ± SE, *N* = 128), which was significantly higher than that of CSB flowers (0.762 ± 0.073 mg, *N* = 29, *P* < 0.001; **see****Supporting Information—Appendix S1**) and CSF flowers (0.844 ± 0.044 mg, *N* = 86, *P* = 0.003; **see****Supporting Information—Appendix S1**).

## Discussion

Our study on *S. miltiorrhiza* demonstrated that shortening of the calyx significantly decreased individual flower longevity, fruit set and seed mass, but did not affect visits by pollinators and nectar robbers, only supporting the hypotheses that persistent calyces may maintain floral longevity and increase plant fitness. Persistent calyces of *S. miltiorrhiza* may provide protection for the growth of flowers after they have emerged fully from the buds and contribute resources (such as carbon, energy, etc.) to the development of fruits and seeds. A previous study in *H. foetidus* revealed that persistent calyx of contributed resources to the development of seeds, and that removing the calyx reduced seed mass rather than seed set (Herrera 2005). Moreover, Vemmos and Goldwin (1994) found that removal of sepals in *M. pumila* significantly reduced fruit set, because sepals are the main photosynthetic parts in this species.

Our calyx manipulations showed that shortening of the calyx significantly reduced floral longevity and female fitness in *S. miltiorrhiza*. Several possibilities may explain this result. First, the general function of the sepals is to protect the flower as it is developing (Griesbach 2002). Calyx sepals can form from a thickened epidermis or can be toughened by sclerenchyma (Willmer 2011). Ryding (2007) found that most species of Lamiaceae have much larger amounts of fibres and similar xylem cells in the calyx tube. Thus, it is likely that campanulate calyces of *S. miltiorrhiza* can protect flowers and fruits from physical damage by heavy rain and strong winds, which are occasional between June and August in the hilly region of northeast Sichuan. Second, the fruits of *S. miltiorrhiza* were always hidden inside the campanulate calyces. The calyx tube has a hairy annulus on the throat inside (Fig. 1E). Therefore, shortening of the calyx may alter the fruit growth micro-environment. Changes in the micro-environmental factors (such as light and temperature) can have adverse effects on fruit development (Polowick and Sawhney 1985; Adams *et al.* 2001; Song *et al.* 2013; Reale *et al.* 2019). Third, calyx sepals are a significant source of assimilates for the developing seeds and fruits (Vemmos and Goldwin 1994; Smillie *et al.* 1999; Salopek-Sondi *et al.* 2000; Aschan and Pfanz 2003; Herrera 2005). Salopek-Sondi *et al.* (2000) found that persistent sepals of *Helleborus niger* are white or rose at anthesis, and the leucoplasts start turning into chloroplasts after pollination. Therefore, chlorophyll-containing sepals may be a major source of assimilates for the developing seeds (Salopek-Sondi *et al.* 2000; Herrera 2005). Similarly, persistent calyces of *S. miltiorrhiza* are purple during flowering, and then gradually turn into green, photosynthetic structures after flowering (Fig. 1F). In this case, the chlorophyll content in the persistent calyces increases, and the persistent calyces may contribute resources (such as carbon and energy) to the development of fruits and seeds. This may explain why shortening of the calyx led to declines in fruit set and seed mass. Previous studies also revealed that removing the calyx had adverse effects on female fitness of plants (Vemmos and Goldwin 1994; Herrera 2005).

Furthermore, our results showed that fruit set and seed set of CSB flowers (calyx shortened at the beginning of blooming stage) were significantly lower than those of CSF flowers (calyx shortened at the beginning of fruiting). The reason could be that the flowers were limited by pollination because of the reduction in individual flower longevity by shortening of the calyx at the beginning of the blooming stage. A previous study suggested that flowers with reduced longevity showed 85 % pollination limitation of fruit set in *Kalmia latifolia* (Rathcke 2003). In our study, the seed sets of control flowers and that of CSF flowers were not significantly different. This may be related to the few ovules (only four ovules) of *S. miltiorrhiza*. However, seed mass of control flowers was significantly higher than that of CSF flowers, suggesting that seed mass was limited by resource availability. This finding is consistent with the previous study by Herrera (2005). Apart from the aspects of functions of calyces discussed above, the clipping of calyx manipulation possibly resulted in some other impacts, for example on the potential physiological/stress responses of flowers, which may also affect individual flower longevity or fruit/seed production.

Our hypotheses of effects on pollinators or nectar robbers were not supported by our manipulation of shortened calyces. According to our observations, the pollinator species *B. breviceps* acquired pollen below the upper lip by biting the tip of the lip. Thus, petals may be more attractive to bumblebee pollinators than campanulate calyces in *S. miltiorrhiza* (Cariveau *et al.* 2004; Willmer 2011). This explains why shortening of the calyx had no effect on bumblebee pollinators. Furthermore, we found that shortening of the calyx did not increase the visitation of nectar robbers. Nectar robbing occurred frequently in *S. miltiorrhiza*, and the flowers presented a high proportion of nectar robbing (88 %) under natural conditions. The reason may be that the calyx tube was shorter than the proboscis of nectar robbers. Although the campanulate calyx covered more than half of the corolla tube, nectar robbers could bite the uncovered part of the corolla tube to obtain nectar. In addition, we observed that bumblebees did not enter the corolla tube to forage for nectar in *S. miltiorrhiza*, which was different from the observations on other species of *Salvia* (Reith *et al.* 2007; Zhang *et al.* 2011). The reason may be that the lower lever arms (staminodes) blocked the flower entrance of bumblebees, or that the corolla tube was too narrow for bumblebees to enter. Although pollen was deposited on the stigma when *B. breviceps* foraged for pollen, the function of the staminal lever was not activated.

*Salvia* consists of over 900 species distributed worldwide (Claßen-Bockhoff *et al.* 2003). The present study constitutes the first report on the effects of shortened calyx on flower growth and fruit/seed development in the *Salvia* genus. We conclude that persistent calyces of *S. miltiorrhiza* may represent adaptive strategies, providing protection for the growth of flowers and increasing female fitness. Persistent calyces are particularly important for fruit/seed development, as we found contributions of persistent calyces to reproductive success at both floral and post-floral stages. However, further studies are needed to provide more details about the exact mechanism behind the functions of persistent calyces.

## Supporting Information

The following additional information is available in the online version of this article—

**Appendix S1.** Scatterplot depicting the association between average seed weight and proportion of seeds produced per aggregate fruit under three treatments.

**Appendix S2.** Raw data sets involved in this study, including nectar robbing, pollination, visitation by pollinators, visitation by nectar robbers, individual flower longevity, fruit set, seed set, seed mass, and seed weight and proportion.

plac004_suppl_Supplementary_Appendix_S1

plac004_suppl_Supplementary_Appendix_S2

## Data Availability

The raw data are available in **Supporting Information—Appendix S2**.

## Notes

**Natural History and Conservation. Chief Editor**: F. Xavier Picó
