# Supplementary material for: Persistent calyces increase floral longevity and female fitness in Salvia miltiorrhiza (Lamiaceae)
Source: AoB Plants. 2022 Jan 27;14(2):plac004. doi: 10.1093/aobpla/plac004 (PMC8903887; doi:10.1093/aobpla/plac004)

Appendix S1. Scatterplot depicting the association between average seed weight (mg) and proportion of seeds produced per aggregate fruit with calyx treatment of control (A), CSB: calyx shortened at the beginning of blooming stage (B), and CSF: calyx shortened at the beginning of fruiting stage (C). The line shown is the simple linear regression. There were more seeds produced by the control and CSF flowers than CSB flowers, and the seed weight of control flowers was higher than that of CSF flowers and CSB flowers.

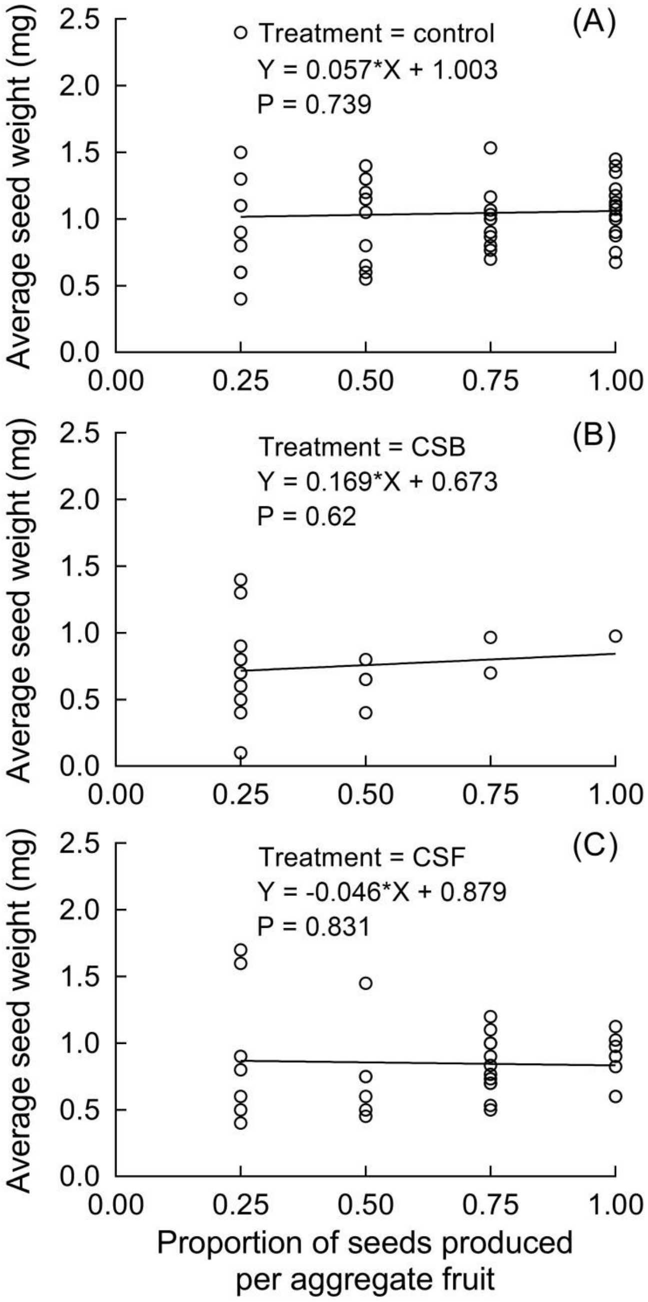

Supplement: plac004_suppl_Supplementary_Appendix_S1 [file plac004_suppl_supplementary_appendix_s1.pdf]
